# Kidney Injury Molecule-1 Is Specifically Expressed in Cystically-Transformed Proximal Tubules of the PKD/Mhm (cy/+) Rat Model of Polycystic Kidney Disease

**DOI:** 10.3390/ijms17060802

**Published:** 2016-05-24

**Authors:** Stefan Gauer, Anja Urbschat, Norbert Gretz, Sigrid C. Hoffmann, Bettina Kränzlin, Helmut Geiger, Nicholas Obermüller

**Affiliations:** 1 Division of Nephrology, III. Medical Clinic, University Hospital Frankfurt, Goethe-University, 60590 Frankfurt, Germany; gauer@em.uni-frankfurt.de (S.G.); helmut.geiger@kgu.de (H.G.); 2 Department of Urology and Paediatric Urology, Philipps-University Marburg, 35043 Marburg, Germany; anja.urbschat@staff.uni-marburg.de; 3 Medical Research Center, University of Heidelberg, 68167 Mannheim, Germany; norbert.gretz@medma.uni-heidelberg.de (N.G.); sigrid.hoffmann@medma.uni-heidelberg.de (S.C.H.); bettina.kraenzlin@medma.uni-heidelberg.de (B.K.)

**Keywords:** Kim-1, polycystic kidney, rat model, proximal tubule, dedifferentiation

## Abstract

Expression of kidney injury molecule-1 (Kim-1) is rapidly upregulated following tubular injury, constituting a biomarker for acute kidney damage. We examined the renal localization of Kim-1 expression in PKD/Mhm (polycystic kidney disease, Mannheim) (cy/+) rats (cy: mutated allel, +: wild type allel), an established model for autosomal dominant polycystic kidney disease, with chronic, mainly proximal tubulointerstitial alterations. For immunohistochemistry or Western blot analysis, kidneys of male adult heterozygously-affected (cy/+) and unaffected (+/+) littermates were perfusion-fixed or directly removed. Kim-1 expression was determined using peroxidase- or fluorescence-linked immunohistochemistry (alone or in combination with markers for tubule segments or differentiation). Compared to (+/+), only in (cy/+) kidneys, a chronic expression of Kim-1 could be detected by Western blot analysis, which was histologically confined to an apical cellular localization in areas of cystically-transformed proximal tubules with varying size and morphology, but not in distal tubular segments. Kim-1 was expressed by cystic epithelia exhibiting varying extents of dedifferentiation, as shown by double labeling with aquaporin-1, vimentin or osteopontin, yielding partial cellular coexpression. In this model, in contrast to other known molecules indicating renal injury and/or repair mechanisms, the chronic renal expression of Kim-1 is strictly confined to proximal cysts. Its exact role in interfering with tubulo-interstitial alterations in polycystic kidney disease warrants future investigations.

## 1. Introduction

Autosomal-dominant polycystic kidney disease (ADPKD) represents the most frequent hereditary renal disease. Worldwide, more than 12 million people are affected [1], and at the age of 60, over 50% have reached end stage renal disease, requiring costly renal replacement therapy. In most cases, mutations in the genes *PKD1* and *PKD2* have been shown to be responsible for the disease [2]. Nevertheless, so far, no promising therapeutic options have been developed. Yet, there is a substantial need to gain more information about effector molecules being chronically active during the progression of the disease, which is characterized by cystic transformation and fibrotic remodelling [3]. In the long term, this might enable the development of pharmacologic interventions that at least retard disease progression. Various rodent models for ADPKD exist. One established model is the PKD/Mhm (polycystic kidney disease, Mannheim) (cy/+) rat (cy: mutated allel, +: wild type allel) in which cyst formation is predominantly observed in proximal tubuli [4,5,6]. In this model, a missense mutation of *Anks6* has been recently discovered as the driving genetic defect associated with polycystic kidney disease; however, its exact cellular function is currently under investigation [7,8].

KIM-1 (kidney injury molecule-1), also termed TIM-1 (T-cell immunoglobulin and mucin domain 1) or HAVCR (Hepatitis A virus cellular receptor 1) or Kim-1 (applying to the rodent orthologue form) has been identified as a crucial proximal tubular cell protein, being markedly upregulated in a huge number of different types of kidney injury as evidenced in animal models and in humans, being virtually not expressed by healthy renal epithelia [9,10,11]. This molecule is a type 1 transmembrane protein with a short cytoplasmic domain possessing tyrosine phosphorylation sites and extracellular mucin- and immunoglobulin-like domains containing *N*- und *O*-glycosylation sites. The glycosylated ectodomain is cleaved by metalloproteinases [12]. Shedding of Kim-1 in the kidney has been proposed to facilitate epithelial regeneration after acute injury [13]. Kim-1 has been shown to regulate immune responses on various levels [14]. Furthermore, Kim-1 can function as a phosphatidylserine receptor on epithelial cells, inducing the removal of apoptotic cells [15]. Kim-1 is a useful biomarker for renal proximal tubular damage [9], and its expression is also associated with renal cell carcinoma, where it is found in carcinoma cells and in the vicinity of tumorous lesions [16]. Kim-1 is not only rapidly upregulated in the proximal tubule during various forms of acute kidney injury, but is also expressed in chronic kidney disease, which is mainly characterized by tubulointerstitial changes and fibrosis [17]. Therefore, we sought to analyze protein expression of Kim-1 in the PKD/Mhm (cy/+) rat.

## 2. Results

**Figure 1:**
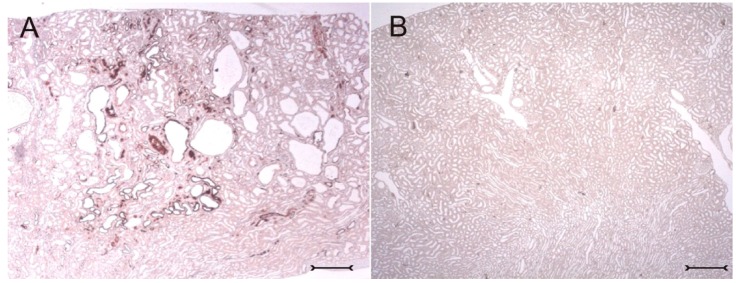
Overview: immunohistochemical expression of kidney injury molecule-1 (Kim-1) in an adult rat kidney cortex of a heterozygous (cy/+) animal (**A**) and a wild type (+/+) non-cystic animal (**B**) using the immunoperoxidase technique. Kim-1-positive tubules including cyst-lining epithelia were only detected in (cy/+), but not in (+/+) kidneys. Bars = 200 µm.

**Figure 2:**
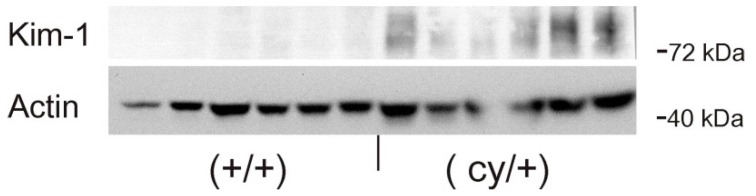
Total protein expression of Kim-1 in adult whole kidney extracts of PKD/Mhm (polycystic kidney disease, Mannheim) rats detected by Western blot. (cy/+): affected kidneys; (+/+): wildtype kidneys. Each lane represents one animal. Blots were reprobed with actin. Kim-1 is only detected in affected kidneys.

In adult (cy/+) kidneys, a distinct immunohistochemical Kim-1 expression can be seen in several tubular, partly cystic structures of the kidney cortex and outer stripe (Figure 1A), whereas no Kim-1 expression is found in the corresponding areas of wildtype (+/+) kidneys (Figure 1B). Western blot analysis confirmed the exclusive expression of Kim-1 in (cy/+) kidney extracts, whereas no bands were identifiable in (+/+) kidneys (Figure 2).

**Figure 3:**
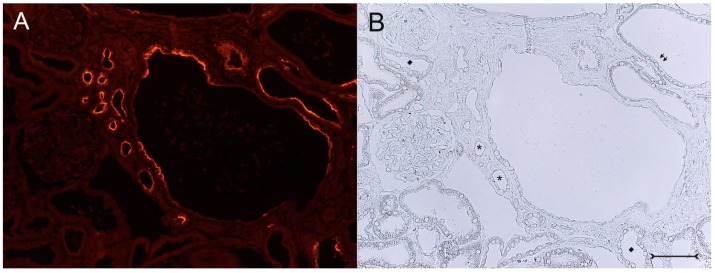
Kim-1 immunohistochemistry with fluorescent staining (**A**), (cy/+) rat kidney cortex; (**B**) corresponding phase contrast image. A mosaic-like expression pattern of Kim-1 in cyst wall epithelia of a large cyst (in the middle) with apically-concentrated labeling is seen; virtually all cells of this cyst are devoid of a brush border and show different morphologies. Small atrophic proximal tubuli (examples are marked with stars in (**B**)) in the vicinity of the central cyst are Kim-1 positive, have markedly lost their brush borders and are surrounded by a compacted, fibrosed interstitium. Distal tubules (examples are marked with diamonds in (**B**)) are devoid of a signal. Parts of the cystic profiles with a maintained brush border (see arrows, upper right in (**B**)) do not express Kim-1. Bar = 75 µm.

**Figure 4:**
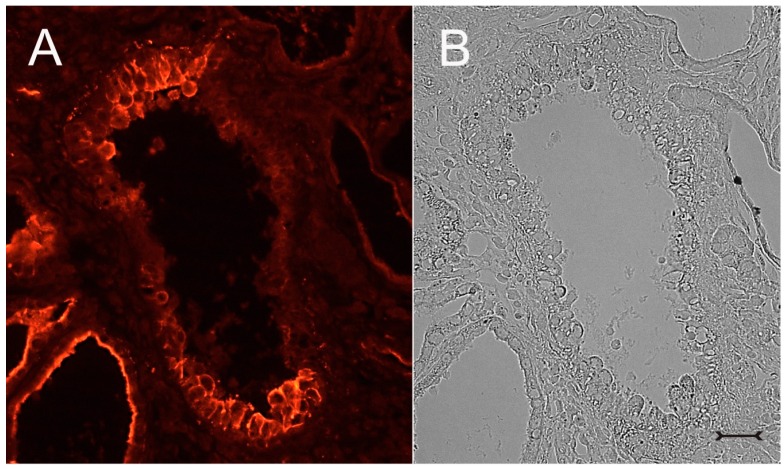
Immunohistochemistry for Kim-1 (**A**) and corresponding phase contrast micrograph (**B**) of a (cy/+) rat kidney cortex. An enlarged cyst with a hyperplastic cyst wall with portions strongly staining for Kim-1 (**A**). In the vicinity, another proximal cyst (bottom left) with continuous apical labeling. Bar = 15 µm.

**Figure 5:**
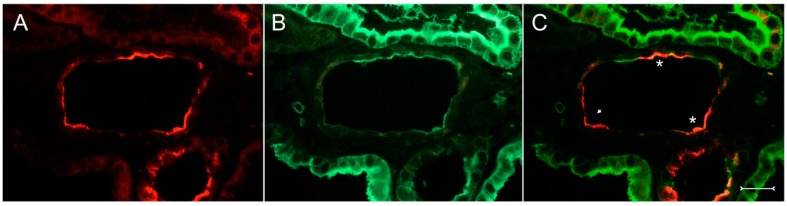
Double fluorescence immunohistochemistry with anti-Kim-1 ((**A**), red) and anti-aquaporin-1 antibodies ((**B**), green); merged view in (**C**). Kidney cortex of a (cy/+) rat. Differentiated proximal tubules do only express aquaporin-1 in their well-developed brush borders (top in (**B**)), while in a cystically-transformed tubule with more flattened cells, Kim-1 expression in the apical cell compartment overlaps with still present, but reduced aquaporin-1 staining (examples are marked with asterisks). Kim-1 is also expressed in cells devoid of aquaporin-1 (see small arrow). At the bottom, a normal-sized tubule also demonstrating partial overlap of Kim-1 and aquaporin-1 staining. Bar = 25 µm.

**Figure 6:**
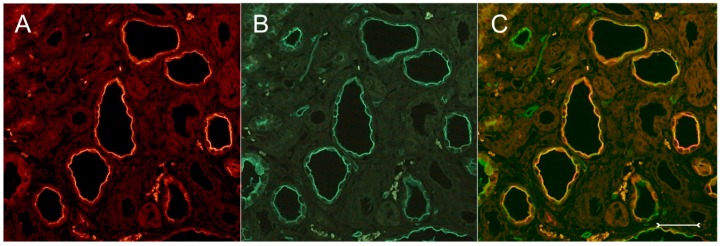
Double fluorescent labeling with anti-Kim-1 ((**A**), red) and anti-aquaporin-1 antibodies ((**B**), green); merged view in (**C**) of cystically-dilated structures which, are descending thin limbs or distal portions of S3 proximal tubules. They show signal overlap in many profiles in the apical rim; view of outer stripe, (cy/+) kidney. Bar = 30 µm.

**Figure 7:**
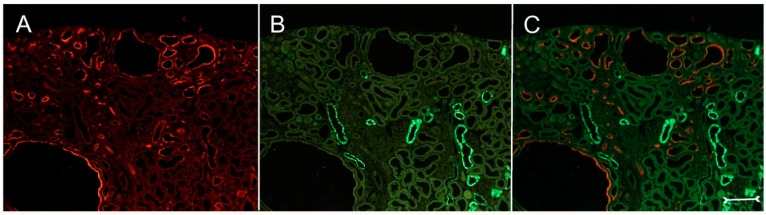
Double labeling experiments with anti-Kim-1((**A**), red) and anti-calbindin-D28k ((**B**), green); merged view in (**C**); in an overview of a (cy/+) kidney cortex. Calbindin-D28k is expressed in distal nephron segments and is not observed in Kim-1-positive profiles or cysts with apically-accented staining. Bar = 150 µm.

A closer view of (cy/+) kidney cortical structures confines Kim-1 labeling to epithelial cells of proximal tubular profiles, which demonstrated a complete loss or massive reduction of their brush borders (Figure 3A,B). Within cystic proximal tubules, Kim-1 is expressed either by scattered or some coherent cells or in agglomerated cell clusters of the cyst wall, while in others, a large majority of cells of a given profile were Kim-1 positive as within atrophic proximal tubuli (Figure 3A). Kim-1 immunoreactivity demonstrated a distinct apical cell distribution (Figure 3A). In addition, Kim-1 is also expressed in parts of hyperplastic cysts with an apical and lateral cell staining pattern (Figure 4A,B). In well-developed brush borders of proximal epithelia expressing the proximal differentiation marker aquaporin-1 (AQP-1), virtually no Kim-1 is expressed, but in neighboring profiles undergoing atrophy, a mixed picture is apparent in double labeling experiments with cells co-expressing both Kim-1 and AQP-1, or only either protein, or even neither protein (Figure 5A–C). Of note, in areas of apparently normal non-dilated proximal tubules, sporadically individual cells exhibit an apical Kim-1 expression (see also Figure 5). No relevant expression of Kim-1 could be revealed in distal nephron segments nor in other regions by carefully analyzing multiple sections. However, some dilated thin descending limb (TDL) profiles, found only in the outer stripe and being AQP-1-positive, were also Kim-1-reactive (Figure 6A–C). These profiles may also represent dilated end portions of S3 proximal tubules. Double labeling experiments with Kim-1 and calbindin-D28k, which labels distal convoluted tubule (DCT) and connecting tubule (CNT) profiles in the cortical labyrinth excluded the expression of Kim-1 in these segments (Figure 7A–C). Thick ascending limbs (TAL) and collecting ducts (CD), identified by their histomorphology, were devoid of signals for Kim-1.

**Figure 8:**
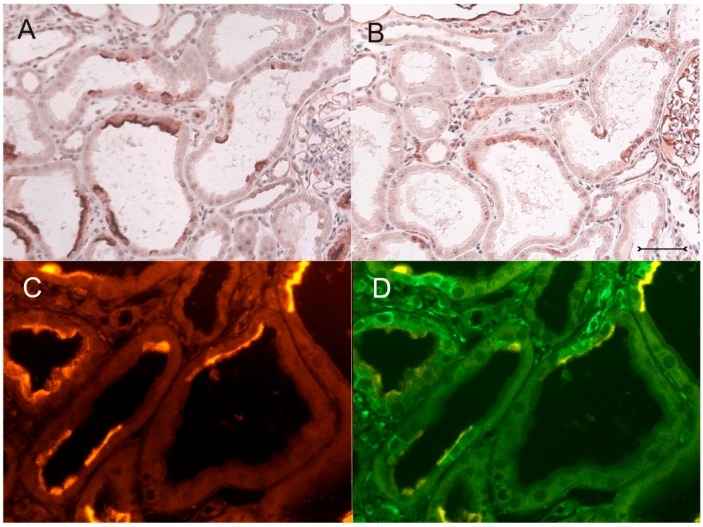

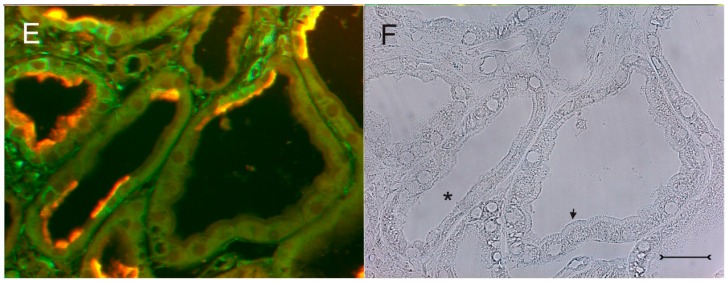
Expression of Kim-1 and vimentin in proximal tubular cysts of the PKD/Mhm (cy/+) rat kidney. (**A**,**B**) Adjacent sections examined by immunohistochemistry (immunoperoxidase labeling) with staining for Kim-1 (**A**) and vimentin (**B**). Tubular profiles in the middle and right of (**A**,**B**) show staining (brown deposits) for both proteins in the same epithelial portions of the cysts. In (**B**) (far right), vimentin staining of glomerular cells exhibits the expected pattern. (**C**–**E**) Fluorescent double labeling with Kim-1 ((**C**), red), vimentin ((**D**), green); merged view in (**E**); phase contrast image in (**F**). Many of the Kim-1 positive cells also express vimentin (an example is marked by an asterisk in (**F**)). The small arrow in (**F**) marks an area of proximal tubular cells with a normal well-expressed brush-border, devoid of any signals. Vimentin is also expressed in interstitial cells (e.g., upper left region in (**D**,**E**)); note: very strong fluorescent signals of Kim-1 are blown-out in (**D**). Bars = 40 µm (**A**,**B**); 30 µm (**C**–**F**).

**Figure 9:**
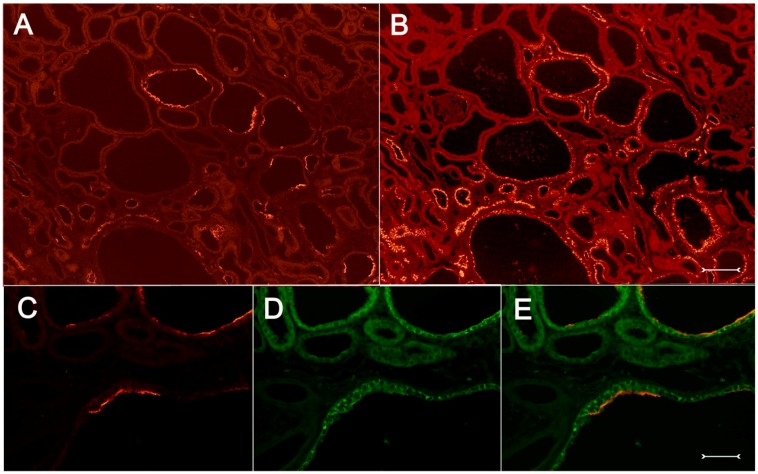
Immunofluorescent expression of Kim-1 and osteopontin in PKD/Mhm (cy/+) rat kidney cortex. In the overview ((**A**,**B**), adjacent sections), Kim-1 (**A**) is only expressed in some cysts, where also osteopontin labeling (**B**) can be detected. Osteopontin staining is found in many more cells and tubular profiles, including distal tubules (**B**). (**C**–**E**) Double fluorescent labeling with anti-Kim-1 ((**C**), red)) and anti-osteopontin antibodies ((**D**), green); merged view in (**E**) in cystic profiles. There is coexpression of both proteins in many cyst wall epithelia, while osteopontin expression is noted in some more cystic cells. Bars = 150 µm (**A**,**B**); 70 µm (**C**–**F**).

In order to further characterize the phenotype of Kim-1-positive cystic cells, double labeling or analysis of adjacent sections was performed with vimentin, a marker for dedifferentiation in proximal tubular cells under diseased conditions [18]. These experiments show that many vimentin-positive profiles in the cystic proximal tubule also coexpressed Kim-1 (Figure 8A–F). Immunohistochemical analysis of Kim-1 and osteopontin, which is upregulated following acute and chronic renal tubular injury [19], identifies many proximal cyst-lining cells as expressing both proteins simultaneously (Figure 9A–E). All experiments performed in corresponding wildtype (+/+) kidneys yielded the expected expression pattern on normal rat kidney; in particular, no Kim-1 immunohistochemical signal could be detected (Figure S1).

## 3. Discussion

Our data demonstrate that Kim-1 protein is chronically expressed in proximal tubules of a rat model resembling human ADPKD. As an important finding, Kim-1 expression is confined to dedifferentiating cystic epithelial cells of the proximal tubule, the main origin of cyst formation in this model.

A previous study also investigated Kim-1 expression in a murine model of polycystic kidney disease. In that model using Pkd2^WS25/−^ mice, however, Kim-1 was found in tubules and cysts of different nephron segments [20]. Yet, similar to our data, detectable amounts of Kim-1 were found in tubular cells, which progressed to a dedifferentiated phenotype. However, both models reveal substantial differences that are not only based on the species background. Whereas in our spontaneous rat mutant model, a defect in the *Anks6* gene is responsible for cystic transformation, in the Pkd2^WS25/−^ mouse, the *Pkd2* gene had been inactivated to generate a *Pkd2* null mutant form, leading to the development of very big cysts in the kidney [21]. Yet, the genetic background, including the genetic modifier, the nephron segments involved and the extent of cyst formation are largely varying between these two models.

The specific expression of Kim-1 also clearly differs from the expression of other injury markers investigated in this rat model previously. Clusterin, a glycoprotein that is expressed by a variety of healthy organs and being also drastically upregulated after diverse tissue injuries, is involved, e.g., in regulation of apoptosis, membrane remodeling and induction of cellular aggregation (reviewed in [22,23]).

Clusterin is strongly induced in the cystic proximal tubule of the (cy/+) rat, but is also massively upregulated in distal tubular segments, including also non-cystic tubules during cyst development and fibrosis [5]. Another phosphorylated acidic glycoprotein, osteopontin (OPN), has attracted considerable attention as a multifunctional cytokine that is also highly expressed following acute and chronic tubular injuries. It has been suggested that it provides anti-apoptotic signals through multiple ligand-receptor interactions to cells [19]. Previous studies showed that OPN is upregulated in this rat model [24], but seems to be expressed more widely in proximal and distal tubular segments compared to Kim-1 (see Figure 9A,B).

With the exception of presumably dilated thin descending limbs (DTL) in the outer stripe, there was no expression of Kim-1 in other tubular nephron segments, nor in other cell types, like fibroblasts or glomerular cells. The apical expression of Kim-1 in these cells, which still did express aquaporin-1 (see Figure 6), is difficult to interpret, since DTL cells are completely different from proximal tubular cells. Furthermore, there were only weak morphological changes as with slight hypertrophy of the putative DTL cells (see Figure 6A–C), but showing direct overlap of Kim-1 and aquaporin-1 in the apical rim. Alternatively, these structures might represent distal parts of the S3 segment of proximal tubules undergoing dilatation and development of a thinned epithelium.

Interestingly, in our study, Kim-1 expression was also detected in vimentin-positive cystic cells. Vimentin has been shown to be expressed in various regenerative tubular lesions, including neoplastic lesions, and the degree of vimentin expression seems to be linked to dedifferentiation [18]. On the contrary, although Kim-1 is expressed mainly in proximal cells having lost their brush borders (which is a classical morphological sign of dedifferentiation), in a few cells, Kim-1 was already expressed together with a reduced apical expression of aquaporin-1 (see Figure 5). This indicates expression of aquaporin-1 in the residual brush border or in the subapical compartment [25]. Undoubtedly, these cells are on the way to dedifferentiation. Thus, in this model, the expression of Kim-1 is detected in cystic cells with a disparate dedifferentiated character. A previous study investigated Kim-1 expression in chronic protein-overload nephropathy, attributing a pathogenetic role to Kim-1 in this disease [26]. They also investigated osteopontin and vimentin in relation to Kim-1 expression and hypothesized that the lack and only scarce cellular overlap in the occurrence of vimentin and Kim-1 argue for a sequential damage-dependent expression of both molecules in proximal tubular cells. However, in our model, colocalization of both proteins was not scarce and was encountered even in smaller cysts; see Figure 8.

Remarkably, in our observations, dedifferentiated cystic cells that did not express Kim-1 were abundant (see, e.g., Figure 3A,B). It is not clear if these dedifferentiated cells expressed Kim-1 previously or not. Our data indicate that Kim-1 might be expressed already during the early phases of cyst development (see again Figure 5 and Figure 7), where smaller cysts already expressed Kim-1. Thereafter, its expression might persist for an unknown time period. Importantly, in this model, cystic cells of proximal tubular origin do not uniformly change into one specific phenotype since cystic wall epithelia exhibit completely different morphologies, such as hyperplastic/proliferative or more flattened or atrophic phenotypes. Of note, Kim-1 expression was detected in both of the hitherto mentioned cell phenotypes. This again points to a heterogeneity of dedifferentiated cystic cell types expressing Kim-1.

From our immunohistochemical data, a clear relation of Kim-1 expression to the development of tubulointerstitial fibrosis in this model was not recognizable, since tubular Kim-1 expression, in fact, was found in areas with advanced tubulointerstitial remodeling, (see, e.g., Figure 3A,B), but not exclusively. Interestingly, in *Ren2* transgenic rat kidneys, overexpressing the mouse renin transgene and leading to tubulointerstitial changes and fibrotic remodeling, Kim-1 is strongly expressed in areas with tubulointerstitial damage. By renin-angiotensin-(RAS)-blockade, a downregulation of Kim-1 expression, as well as an amelioration of tubulointerstitial damage was noted, thus pointing to a contribution of Kim-1 to a RAS-mediated tubular damage [27]. The profibrotic actions of Kim-1 after prolonged exposure have also been demonstrated in a row of experiments showing that, *i.e.*, mutant, truncated Kim-1 led to an improvement of experimental renal fibrosis [15].

Depending on the mode of renal tubular damage (acute *vs.* chronic), Kim-1 may act differently in proximal tubular segments [28]. In acute kidney injury, the vulnerable S3 segment is mainly concerned and the site where Kim-1 acutely accomplishes repair processes. By contrast, in chronic tubulointerstitial disease, stimulation of Kim-1 takes place anywhere in the entire proximal tubule. In the Hannover Spraque-Dawley Han:SPRD (cy/+) rat and the PKD/Mhm substrain, proximal cysts develop mainly, but not exclusively in the S2 segment of the proximal tubule [4,5]. It cannot be excluded that in this model of chronic tubular damage, Kim-1 is only a pure indicator of injury or might only be involved in cell adhesion, as suggested earlier [10]. However, it can be hypothesized that the role of Kim-1 in cysts of the PKD/Mhm rat appears to be more particular than that, e.g., of osteopontin or clusterin, which are considered to exhibit a more classical response to injury. The significance of Kim-1 upregulation with regard to the petrogenetically-relevant *Anks6* mutations in this disease model remains another issue of interest.

Novel data are attributing an important role to Kim-1 as a scavenger receptor relevant in autophagy [15,29]. These results open new avenues for addressing the role of Kim-1 in the course of chronic kidney disease, characterized by interstitial remodeling and fibrosis, the latter condition being an unsolved problem in polycystic kidney disease. Increasing evidence supports the significance of autophagy in the pathogenesis of multiple renal diseases. Importantly, the mTOR (mammalian target of rapamycin) pathway as the crucial inhibitor of autophagy is upregulated in renal diseases, including ADPKD (for a review, see [30]). However, whether blockade or promotion of autophagy could halt cyst development and fibrosis is not clear, as with the function of Kim-1 in this context. Future work will have to refine these possibilities.

## 4. Experimental Section

### 4.1. Animals

The (cy/+) rats were derived originally from the Han:SPRD rat strain [31]. They have been inbred now for largely over 40 generations in the animal care facility of the Medical Research Center of the University of Heidelberg in Mannheim, Germany, and have therefore been registered as a substrain termed PKD/Mhm (for polycystic kidney disease, Mannheim, inbred strains of rats) [7,32]. Offspring of the inbred colony, including (cy/+), as well as (+/+) littermates, were kept under standard laboratory conditions (12-h light cycle, 55% ± 5% humidity, 20 ± 2 °C room temperature) in the animal care facility in Mannheim, Germany. All animals were allowed free access to tap water and rat chow containing 19% protein. Only male wildtype and heterozygous rats (aged 8–12 weeks) were used for subsequent experiments. At this stage, cyst development has already progressed and shows considerable histomorphological alterations in (cy/+) animals [4,5]. Experiments were conducted in accordance with the German Animal Protection Law and were approved by the local authorities (Aktenzeichen: G 78/07 and I 15/10).

### 4.2. Tissue Preparation

For optimal preservation of renal tissue morphology, rats were subjected to retrograde perfusion-fixation with paraformaldehyde (PFA) through the intrarenal abdominal aorta, as described earlier [5]. In brief, rats were deeply anesthetized by an intramuscular injection of ketamine (100 mg/kg) and xylazine (5 mg/kg) and perfused with 2% freshly-depolymerized paraformaldehyde (PFA) in phosphate-buffered saline (PBS), pH 7.4, for 3 min at a pressure level of 180 mmHg. Thereafter, right kidneys were removed, carefully cut into coronary slices and fixed again in the same fixative overnight. Tissues were then embedded in paraffin for subsequent histological examination by immunohistochemistry and hematoxylin/eosin (HE) staining. Slides were viewed by a Leica DM RB microscope using a Leica DFC 450 camera (Leica, Wetzlar, Germany) for documentation.

To obtain fresh renal tissue for subsequent protein preparation prior to perfusion-fixation, left renal pedicles were clamped, and the native kidneys were rapidly removed, dissected free, weighed and shock frozen in liquid nitrogen prior to storage at −80 °C. To verify the cystic phenotype at that stage, respective kidney poles were investigated histologically by HE staining.

### 4.3. Immunohistochemistry

Paraffin-embedded renal tissues from at least 5 (cy/+) and (+/+) male littermates were analyzed. Sections 4-µm thick were deparaffinized, washed in bidistilled water and finally equilibrated in 10 mM citric acid, pH 6.0, for 10 min. For antigen retrieval slides were heated in a microwave oven at 500 W for 5 min followed by a 10-min treatment at 250 W. After the slides were allowed to cool down to room temperature and rinsed three times with Tris-buffered saline (TBS), they were incubated in blocking solution (containing 50% fetal bovine serum in TBS) for 30 min in a moist chamber. The sections were then incubated with one or two primary antibodies (in the case of fluorescent double labeling experiments). If peroxidase-labeled antibodies were used for visualization, slides were treated with 0.3% H_2_O_2_ in methanol for 10 min prior to the antigen retrieval step and were later processed using the VectaStain ABC Kit^®^ (Vector Labs. Inc., Burlingame, CA, USA).

The following primary antibodies, including nephron segment-specific markers and cell differentiation markers, were used: a polyclonal goat anti-rat Kim-1 antibody, (AF3689, R & D Systems GmbH, Wiesbaden, Germany), at a concentration of 2 µg/mL; a rabbit polyclonal anti-human aquaporin-1 (AQP-1) antibody (H-55, Santa Cruz Biotechnology Inc., Santa Cruz, CA, USA) recognizing rat AQP-1, at a concentration of 2 µg/mL; a mouse monoclonal anti-calbindin-D28k antibody, developed against bovine kidney calbindin (clone cb-955, Sigma Aldrich, St. Louis, MO, USA) with broad species reactivity, diluted 1:500; a mouse monoclonal anti-mouse osteopontin antibody (AKm2A1, Santa Cruz Biotechnology Inc., Santa Cruz, CA, USA) recognizing rat osteopontin, concentrated 1 µg/mL; and a mouse monoclonal anti-porcine vimentin antibody (V9, Santa Cruz Biotechnology Inc., Santa Cruz, CA, USA) recognizing rat vimentin (final concentration: 0.5 µg/mL). Aquaporin-1 is constitutively expressed by proximal tubules and cells of the thin descending limb (DTL) in the rat [25]. Calbindin-D28k is expressed by distal convoluted tubule (DCT) and connecting tubule (CNT) cells [33]. In healthy adult rat kidney, osteopontin is only detected in DTL cells [34]; vimentin expression has been attributed to glomerular cells and blood vessels and a few interstitial cells [35]. Subsequent signal detection was accomplished by using fluorescent- or horseradish-labeled secondary antibodies.

After incubation of the sections with the primary antibodies for 1 h at 37 °C and then overnight at 4 °C in a moist chamber, sections were washed three times with TBS and incubated with either a Cy3-labeled donkey anti-goat antibody, diluted 1:200 (Jackson ImmunoResearch, Newmarket, Suffolk, UK), a Cy3-labeled goat anti-mouse or a Cy2-labeled donkey anti-mouse antibody, diluted 1:100 (Jackson ImmunoResearch, Newmarket, Suffolk, UK), or a fluorescein isothiocyanate-coupled mouse anti-rabbit antibody, diluted 1:100 (Sigma-Aldrich, Munich, Germany) or in combination for double immunohistochemistry for 1 hour in the dark, washed and mounted in Moviol.

To confirm fluorescent immunohistochemical signals of Kim-1, detection using the biotin-streptavidin-peroxidase complex method, including visualization with 3-amino-9-ethyl carbazole (AEC), was applied on some sections. Moreover, for comprehensive analysis and to ultimately exclude the binding interference of primary antibodies, adjacent sections were analyzed to compare renal expression sites of marker proteins in relation to Kim-1. For control experiments, the respective normal serum or blocking solution alone was applied instead of the primary antibody, and the slides were processed as described. No specific staining was obtained under these conditions.

### 4.4. Western Blotting

Individual tissue (left kidney of (cy/+) or (+/+) rats) was first ground in liquid nitrogen and then homogenized in sample buffer containing sodium laureth sulfate (SDS) (62.5 mmol/L Tris-HCl, pH 6.8, 4% SDS, 10% glycerol) using a potter homogenizer. Protein content was determined by a BCA-assay (Pierce, Rockford, IL, USA). After a 10-min incubation at 95 °C, 10 µg of total cellular protein from each kidney were loaded onto a 7.5% SDS-polyacrylamide-gel. After electrophoresis, proteins were transferred to a polyvinylidene difluoride membrane using a semidry blotter and a transfer buffer consisting of 48 mM Tris, 39 mM glycine, 0.0375% SDS and 20% methanol. The blots were blocked with 5% fat-free dry milk in Tris-buffered saline, 0.1% Triton X-100, 0.05% Tween 20 (TTBS), for 2 h and incubated with the goat anti-rat Kim-1 antibody (AF3689, R&D Systems, 1 µg/mL) overnight at 4 °C. After washing with TTBS, the blots were incubated with a rabbit anti-goat Ig antibody coupled to horseradish peroxidase (Amersham, Braunschweig, Germany) diluted 1:5000 in TTBS. After washing, the enzyme was visualized using an ECL™ Prime Western Blotting System (Amersham, Braunschweig, Germany). No signal was observed when nonspecific goat Ig was used as the primary antibody. Following Western blot detection, blots were incubated with 50 mM Tris-HCl (pH 7), 2% sodium laureth sulfate, 50 mM dithiothreitol at 70 °C for 30 min, washed with TTBS and reprobed with a mouse anti-actin antibody (Chemicon, Temecula, CA, USA).

## 5. Conclusions

Kim-1 is expressed in dedifferentiating renal proximal tubules during cystogenesis in experimental polycystic disease of the rat. Whether Kim-1 is involved in a cellular response to injury or whether it participates in disease progression e.g., via affecting autophagy has to be established.

## Supplementary Materials

Supplementary materials can be found at http://www.mdpi.com/1422-0067/17/6/802/s1.
