# Supplementary material for: Kidney Injury Molecule-1 Is Specifically Expressed in Cystically-Transformed Proximal Tubules of the PKD/Mhm (cy/+) Rat Model of Polycystic Kidney Disease
Source: Int J Mol Sci. 2016 May 24;17(6):802. doi: 10.3390/ijms17060802 (PMC4926336; doi:10.3390/ijms17060802)
Supplement: Supplementary file 1 [file ijms-17-00802-s001.pdf]

Supplementary Materials: Kidney Injury Molecule-1 is Specifically Expressed in Cystically-Transformed Proximal Tubules of the PKD/Mhm (cy/+) Rat Model of Polycystic Kidney Disease

Stefan Gauer, Anja Urbschat, Norbert Gretz, Sigrid C. Hoffmann, Bettina Kränzlin, Helmut Geiger and Nicholas Obermüller

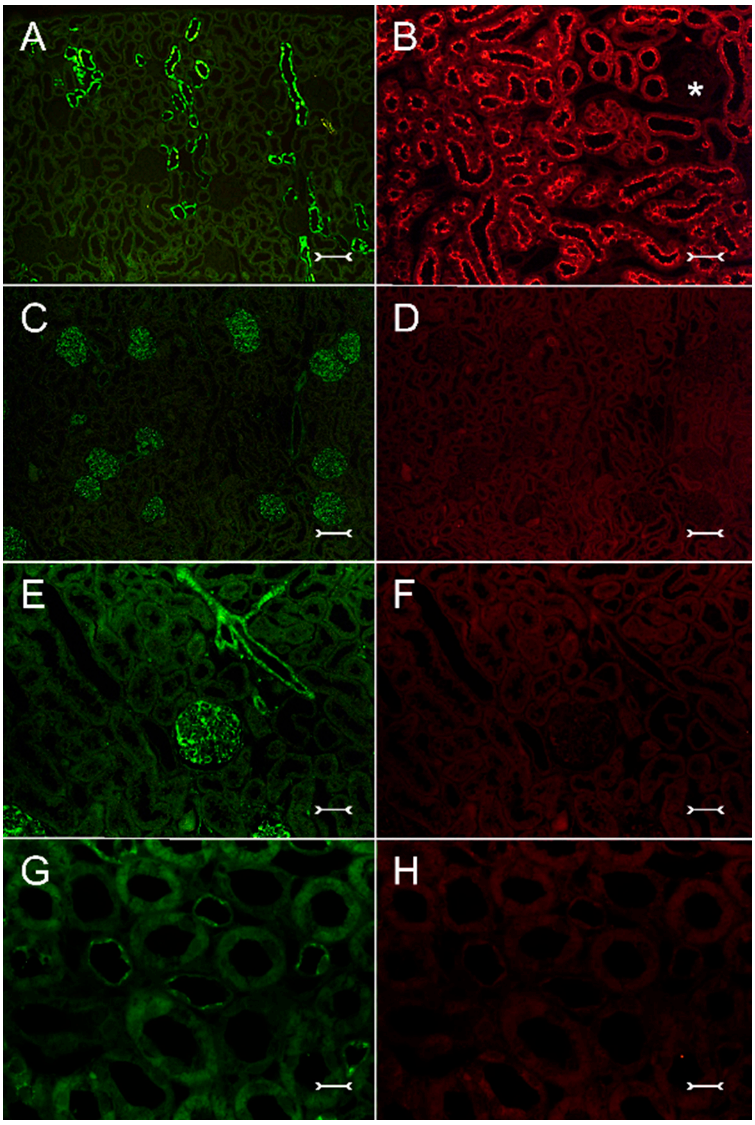

Figure S1. Immunofluorescent staining of calbindin-D28k (**A**); aquaporin-1 (**B**); vimentin and Kim-1 (**C**–**F**); osteopontin and kidney injury molecule-1 (Kim-1) (**G**,**H**) in wildtype polycystic kidney disease, Mannheim (PKD/Mhm) (+/+) rats. (**C**–**H**) represent double labeling experiments; (**A**) Calbindin-D28k (green) is expressed in cells of connecting tubule (CNT) and distal convoluted tubule (DCT) profiles of outer and inner cortex; (**B**) Aquaporin-1 (red) is expressed in the brush border of almost all proximal tubules in the kidney cortex view. Star marks a glomerulus; (**C**) Vimentin (green) is strongly expressed in virtually all glomeruli of the kidney cortex, whereas no Kim-1 staining is detectable by double labeling on the same section (**D**); (**E**) Detailed view of vimentin expression (green) in cells of a glomerulus in the middle and in the vasculature exhibiting a striped pattern (upper right) while Kim-1 is not expressed in any structure (**F**); (**G**) Detailed view of outer medulla, osteopontin (green) shows delicate expression in cells of dilated thin descending limbs (DTL) profiles while Kim-1 is devoid of a signal as shown in (**H**); Bars = 100 µm (**A**,**C**,**D**); 50 µm (**B**,**E**,**F**); 38 µm (**G**,**H**).
